# Magnetic Ionic Liquid: A Multifunctional Platform for the Design of Hybrid Graphene/Carbon Nanotube Networks as Electromagnetic Wave-Absorbing Materials

**DOI:** 10.3390/molecules30050985

**Published:** 2025-02-20

**Authors:** Jean C. Carelo, Bluma G. Soares, Debora P. Schmitz, Ruan R. Henriques, Adriana A. Silva, Guilherme M. O. Barra, Vitoria M. T. S. Barthem, Sebastien Livi

**Affiliations:** 1 Centro de Tecnologia, COPPE-PEMM, Universidade Federal do Rio de Janeiro, Bl. F, Rio de Janeiro 21941-598, Brazil; jeancarelo@metalmat.ufrj.br; 2 Centro de Tecnologia, Instituto de Macromoléculas, Universidade Federal do Rio de Janeiro, Bl. J, Rio de Janeiro 21941-598, Brazil; dehschmitz@gmail.com (D.P.S.); ruanhenriara@gmail.com (R.R.H.); 3 Centro de Tecnologia, Escola de Química, Universidade Federal do Rio de Janeiro, Bl. E, Rio de Janeiro 21941-909, Brazil; anjosadriana@eq.ufrj.br; 4 Departamento de Engenharia Mecânica, Universidade Federal de Santa Catarina, Florianópolis 88040-900, Brazil; guiga@emc.ufsc.br; 5 Centro de Tecnologia, Instituto de Física, Universidade Federal do Rio de Janeiro, Bl. A, Rio de Janeiro 21941-909, Brazil; vitoria@if.ufrj.br; 6 Université de Lyon, CNRS, Université Claude Bernard Lyon 1, INSA Lyon, Université Jean Monnet, UMR 5223, Ingénierie des Matériaux Polymères, CEDEX F-69621 Villeurbanne, France

**Keywords:** epoxy resin, magnetic ionic liquid, graphene nanoplatelet, hybrid filler, electromagnetic interference shielding effectiveness, microwave-absorbing properties

## Abstract

Magnetic ionic liquid (MIL) based on alkyl phosphonium cation was used as a curing agent for developing epoxy nanocomposites (ENCs) modified with a graphene nanoplatelet (GNP)/carbon nanotube (CNT) hybrid filler. The materials were prepared by a solvent-free procedure involving ball-milling technology. ENCs containing as low as 3 phr of filler (GNP/CNT = 2.5:0.5 phr) exhibited electrical conductivity with approximately six orders of magnitude greater than the system loaded with GNP = 2.5 phr. Moreover, the use of MIL (10 phr) resulted in ENCs with higher conductivity compared with the same system cured using conventional aliphatic amine. The filler dispersion within the epoxy matrix was confirmed by scanning electron microscopy (SEM) and transmission electron microscopy (TEM). The electromagnetic interference shielding effectiveness (EMI SE), evaluated in the X- and Ku-band frequency range, revealed a great contribution of the absorption mechanism for the ENC containing the hybrid filler and cured with MIL. Moreover, the best microwave-absorbing response was achieved with the ENC containing GNP/CNT = 2.5/0.5 phr, and cured with ML, which a minimum RL of −23.61 dB and an effective absorption bandwidth of 5.18 GHz were observed for thickness of 1.5 mm. In summary, this system is a promising material for both civilian and military applications due to its simple and scalable nanocomposite preparation method, the lightweight nature of the composites resulting from the low filler content, the commercial availability and cost-effectiveness of GNP, and its high electromagnetic wave attenuation across a broad frequency range.

## 1. Introduction

Electromagnetic interference (EMI) poses a significant challenge in modern electronic devices and systems, creating a strong demand for advanced materials with enhanced electromagnetic wave-absorbing properties [1,2,3]. Conductive polymer composites are commonly used in this context due to their low cost, lightweight nature, ease of processing, and versatility in production with different designs. Moreover, the conductivity and dielectric/magnetic properties of these composites can be easily tailored by selecting suitable fillers and polymer matrices, as well as optimizing the dispersion process [1,4,5,6].

Epoxy matrices are versatile thermosetting materials, widely used in applications such as coatings, adhesives, fiber-based composites, and electronic devices [7]. For electromagnetic wave (EMW) absorption applications, it is necessary to add conductive and dielectric/magnetic fillers that are able to interact with the incident EMW. In this context, carbon-based fillers, such as carbon nanotube (CNT) and graphene-related materials (graphene nanoplatelets (GNPs), expanded graphite (EG), reduced graphene oxide (rGO) and thermally reduced graphene oxide (TRGO)) have been extensively explored for EMI shielding/absorbing applications [8,9,10,11,12,13,14,15]. Among these graphene materials, GNP is a commercially viable product with relatively low cost, making it ideal for large-scale production. The effectiveness of GN-based epoxy systems for EMW shielding/absorption materials has been discussed in several reports and reviews [10,12]. GNPs are available with varying number of layers and surface areas, which significantly influence the electrical, dielectric, and EMI shielding/absorbing properties of the corresponding composites [16,17].

For example, Kargar et al. [18] blended commercially available few-layer graphene with epoxy resin and achieved a total EMI shielding effectiveness (EMI SE) of 45 dB for the composite 1 mm thick, containing 50 wt% filler content. Liang et al. [19] reported an EMI SE of 21 dB for the epoxy/rGO composite with 3 mm thickness and 15 wt% of filler. Ahmad et al. [20] prepared epoxy composites with 5 wt% of rGO and 6 mm thickness, observing EMI SE ranging from 5 to 25 dB as the microwave frequency varied between 8 and 12 GHz. The attenuation of the radiation through the absorption of epoxy/GNP nanocomposites has also been studied using the reflection loss (RL) parameter. For instance, Min et al. [21] prepared epoxy/GNP composites via a solution method and reported an RL value of −38 dB for a composite containing 2 wt% GNP with a thickness of 1.5 mm. Wang et al. [22] observed a minimum RL of −14.5 dB at 18.9 GHz for epoxy/GNP composite with 15 wt% GNP loading. Silva et al. [23] reported an RL value of −11.7 dB at 8.75 GHz with 0.9 vol% of GNP. These varying results can be attributed to differences in the GNP suppliers, which affect factors such as exfoliation degree, surface area, number of layers, and the synthetic procedure for preparing epoxy/GNP composites.

GNP faces significant dispersion challenges due to strong van der Waals forces and π-π stacking interactions. As a result, relatively large amounts of GNP (10–50 wt%) are often required to achieve EMI SE greater than 10 dB, corresponding to 90% of EMW attenuation [12,17,24]. High filler content, however, can lead to poor mechanical performance, processing difficulties, and increased costs. To address these issues, various strategies have been explored. Recent studies have shown that combining GNP with CNT can significantly enhance the electrical conductivity [25], mechanical strength, rheological behavior, and thermal properties of epoxy composites [26,27,28,29,30,31,32,33,34,35]. The GNP/CNT hybrid prevents self-aggregation and re-stacking of graphene layers, improving the electrical response due to the formation of a three-dimensional percolated structure, where the flexible CNTs act as bridges between the planar GNP nanoplatelets [29,36]. These hybrid fillers offer an economical solution for developing conducting polymer composites with low filler content [34].

Several studies have reported on the EMI shielding/absorbing properties of epoxy composites containing hybrid fillers, including graphene-related materials (GNP, rGO, etc.) and CNT. For example, Kang et al. [37] prepared rGO/single-walled carbon nanotube hybrids by chemical-grafting CNT onto GO, followed by reduction. The addition of 15% of this hybrid to an epoxy matrix resulted in an EMI SE of approximately 71 dB at 12 GHz. Han et al. [38] grafted vertical edge-rich graphene onto a CNT sponge and blended it with epoxy resin, achieving an EMI SE of 46.9 dB in the X-band (8–12 GHz) with a thickness of 1 mm. Lee et al. [39] mixed commercial GNP and CNT with epoxy resin in a three-roll mill, reporting an EMI SE of 27 dB at 100 MHz with a 50:50 GNP/CNT hybrid (10 wt% total filler). Huangfu et al. [40] prepared epoxy composites with varying amounts of CNT/thermally annealed graphene aerogel and reported an EMI SE of approximately 35 dB in the 8–12 GHz frequency range. Zhang et al. [41] synthesized reduced graphene/CNT oxides from mixed powders of expanded graphite and CNT, achieving an RL of −61.8 dB with a 1 wt% hybrid in an epoxy network with a thickness of 1.9 mm. An epoxy/GNP/CNT hybrid with foamed structure was also developed by Liu et al. [42], obtaining RL of −20 dB. Yazdi et al. [43] reported an EMI SE value of 14 dB at 8.2 GHz for an epoxy composite containing TRGO/CNT (2.5:2.5 wt%), and 11 dB for epoxy with 8 wt% CNT.

Non-covalent functionalization agents can improve the dispersion of carbon nanomaterials without affecting their electrical conductivity. In this context, ionic liquids (ILs) play a crucial role, as they can interact with the carbon surface via π-cation and van der Waals forces [44,45]. Several studies have used ILs to enhance the dispersion of CNT in epoxy matrices [46,47,48,49], although their use in GNP-based systems remains underexplored. Imidazolium-based ILs with chloride as the counteranion were used to modify GNP [50] and GO [51], enhancing the thermal and mechanical properties of epoxy composites. Liu et al. [52] achieved better dispersion of GNP in an epoxy system by pre-treating it with 1-aminopropyl-3-butylimidazolium hexafluorophosphate. The enhancement of EMW absorption properties in epoxy/GNP systems has been reported by using GNP functionalized with 1-butyl-3-methyl-imidazolium bis(trifluormethylsulfonyl) imide [23]. Additionally, the dual role of magnetic phosphonium- and imidazolium-based ILs as curing agents for epoxy resin and additives for enhancing EMW-absorbing properties has been demonstrated [53]. The presence of 10 phr (9.1 wt%) of magnetic phosphonium IL resulted in RL values of around −13 dB (≈96% of EMW attenuation) at 9.5 GHz.

Building on these foundational studies, the present work aimed to investigate the multifunctional effects of phosphonium-based magnetic ionic liquid (MIL) in an epoxy system containing GNP/CNT hybrids. As discussed in several reports and reviews, the combination of magnetic and dielectric materials in a composite can enhance the microwave absorption performance [54,55,56,57,58]. Although the magnetic ionic liquid exhibits relatively low magnetic loss, its combination with carbonaceous fillers can improve electrical conductivity and enhance microwave absorption properties. Thus, the advantages of using MIL as the curing agent were based on its multifunctionality, that is, its ability to disperse the carbonaceous filler, the ability of curing the epoxy resin with relatively low amount of material, when compared with conventional aliphatic amine and anhydride as hardeners, and the introduction of some magnetic characteristics, which improve the microwave absorption properties. By leveraging the properties of magnetic ILs and the synergistic effects of GNP/CNT hybrids, this study seeks to enhance electrical conductivity, mechanical strength, rheological behavior, thermal properties, and EMI shielding/absorption effectiveness, paving the way for advanced materials in EMI applications.

## 2. Results and Discussion

### 2.1. AC Conductivity of the Epoxy Composites

**Figure 1:**
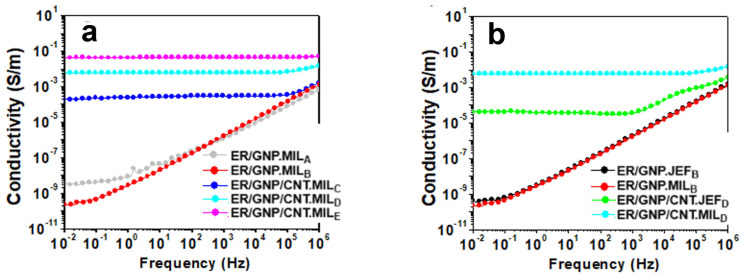
AC conductivity (σ_AC_) as a function of frequency for ER composites. (**a**) ER/GNP and ER/GNP/CNT cured with MIL; (**b**) comparison between the composites cured with MIL and with Jeffamine D230.

The AC conductivity (σ_AC_) versus frequency curves for ER/GNP and ER/GNP/CNT hybrids cured with magnetic ionic liquid (MIL) are shown in Figure 1a. The composite containing 2.2 wt% of GNP (ER/GNP.MIL_B_) exhibits a typical insulating behavior, characterized by the linear relationship between σ_AC_ and frequency. Increasing the amount of GNP to 8.3 wt% resulted in an increase in σ_AC_ by one order of magnitude and the presence of a very small σ_DC_ plateau at low frequency, but the insulating behavior was still observed. This result suggests that the commercial GNP grade used in this work was not well exfoliated, and its dispersion within the epoxy matrix was insufficient to form a conductive pathway, even at 8.3 wt% of GNP. A similar behavior was reported in the literature for epoxy systems modified with 10 wt% GNP [16].

As discussed in the Introduction, the combination of CNT with GNP leads to a synergistic effect in terms of electrical conductivity because the fillers can interact with each other, preventing aggregation and re-stacking of graphene layers [29,31,36]. Therefore, the impact of different CNT amounts on the conductivity of the ER/GNP.MIL systems containing 2.2 wt% of GNP was investigated. As observed in Figure 1a, the addition of just 0.2 wt% of CNT resulted in a significant increase in conductivity, by around six orders of magnitude, reaching a value of 2 × 10^−4^ S/m at low frequency. Another notable feature is the presence of a σ_DC_ plateau up to a frequency of 10^5^ Hz, which characterizes the material as conductive. As stated in various reports, GNP presents a higher tendency of agglomeration and re-stacking than CNT, decreasing the chance in creating the conductive network structure [39,59,60]. On the other hand, CNT, with its one-dimensional structure and higher aspect ratio, can be more easily dispersed in a polymeric matrix. Thus, the addition of a low amount of CNT exerted a positive influence on the electric conductivity of the ER/GNP composites. The CNTs help with the dispersion of GNPs by acting as a bridge between the GNP platelets, facilitating the deagglomeration of GNP and promoting the formation of a three-dimensional percolated structure that enhances the electrical response. This result is interesting because a high conductivity value can be achieved with only 2.4 wt% of filler, which is advantageous for processing the material and reducing cost. Increasing the CNT content further increased the conductivity.

To highlight the dual role of the magnetic ionic liquid (MIL) as both hardener and dispersing agent for the filler, ER/GNP and ER/GNP/CNT were also cured with the conventional aliphatic amine, Jeffamine^®^D230. For this study, the proportions of GNP/CNT were kept as 2.5/0.5 phr because of the acceptable electrical conductivity and the superior microwave-absorbing properties achieved with this composition. Furthermore, the viscosity was not so high as to affect the processability and dispersion of the fillers. Figure 1b compares the results. The nature of the curing agent did not affect the conductivity of the ER/GNP binary system. Both ER/GNP.MIL_B_ (2.2 wt% of GNP) and ER/GNP.JEF_B_ (1.9 wt% of GNP) exhibited similar insulating behavior.

It is important to point out that the ER composites containing the GNP/CNT hybrid filler presented higher conductivity than those loaded with only GNP as the filler, regardless of the nature of the curing system. By comparing the two systems with GNP/CNT hybrid filler, the one cured with MIL presented conductivity value by more than two orders of magnitude higher than the system cured with Jeffamine. The former composite contained a slightly larger amount of GNP (GNP/CNT = 2.2: 0.4 wt%) than that cured with Jeffamine (GNP/CNT—1.8:0.4 wt%), which may be one factor that contributed for its higher conductivity value. However, the difference in the amount of GNP was not excessive. Thus, one can suggest that the MIL interacts with the carbonaceous fillers through cation–π interactions, thus enhancing the dispersion of the fillers, which favors the formation of the three-dimensional networked structure responsible for the increase in electrical conductivity. This characteristic is also suggested from XRD experiments, discussed lately.

### 2.2. Rheology of Uncured Epoxy Dispersions

**Figure 2:**
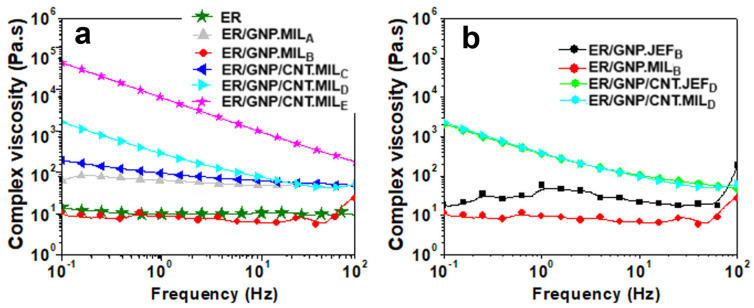
Complex viscosity as a function of frequency for ER composites. (**a**) ER/GNP and ER/GNP/CNT cured with MIL; (**b**) comparison between the composites cured with MIL and with Jeffamine D230.

Rheological analysis is commonly employed to assess the dispersion behavior of fillers within a polymer matrix. Figure 2a presents the complex viscosity (η*) as a function of frequency, measured at 25 °C, for the epoxy systems before curing. The η* of neat epoxy remained constant across frequencies, characteristic of a Newtonian fluid. A similar trend and η* value were observed for the ER/GNP.MIL_B_ (2.2 wt% GNP) system. When the GNP content was increased to 8.33 wt%, η* also increased, yet the system maintained its Newtonian behavior. The incorporation of CNT into the ER/GNP.MIL system led to a rise in η* at low frequency, with the effect becoming more pronounced as the CNT content in the hybrid increased. Moreover, the composites with hybrid fillers exhibited shear thinning behavior, which became more prominent with increasing CNT content [61]. This behavior is attributed to the formation of a three-dimensional physical network of fillers within the epoxy matrix, induced by interactions between the fillers, thereby confirming better filler dispersion within the matrix [62]. Figure 2b compares η* versus frequency for the uncured systems containing MIL and Jeffamine. In the case of the ER/GNP system, replacing MIL with Jeffamine resulted in a slight increase in η*, suggesting a plasticizing effect of the ionic liquid. However, for the ER/GNP/CNT system, the η* remained similar. We hypothesize that MIL interacts with CNT, leading to an increase in viscosity, which is counterbalanced by the plasticizing effect of the MIL. Consequently, the interactions between MIL and CNT prevent a decrease in viscosity, resulting in the ER/GNP/CNT/MIL composite exhibiting a viscosity similar to that of the ER/GNP/CNT/Jef composite.

Figure S1 illustrates the relationship between the elastic and viscous moduli, revealing a marked variation in the viscoelastic behavior of the composites. The straight line in the graph represents the points where the elastic modulus (G′) equals the viscous modulus (G″). Above this line, known as the equimodular line, the elastic component dominates the material’s behavior, while below it, viscous mechanisms have a greater influence on the sample’s properties [63]. The Han Plot, or modified Cole–Cole plot, shows the correlation between CNT concentration and viscoelastic behavior. Composites with a low CNT concentration lie below the percolation line, indicating a predominance of viscous behavior. On the other hand, at a CNT concentration of 0.4 wt%, rheological percolation occurs, as shown by the alignment of the curves with the equimodular line, suggesting the formation of networks that balance both elastic and viscous properties. Notably, composites with higher CNT content, such as ER/GNP/CNT.MIL_E_, exhibit a dominance of elastic properties, reflecting greater material rigidity. This underscores the significant impact of CNT concentration on the formation of the physical network.

### 2.3. XRD and Morphology of Hybrid System

**Figure 3:**
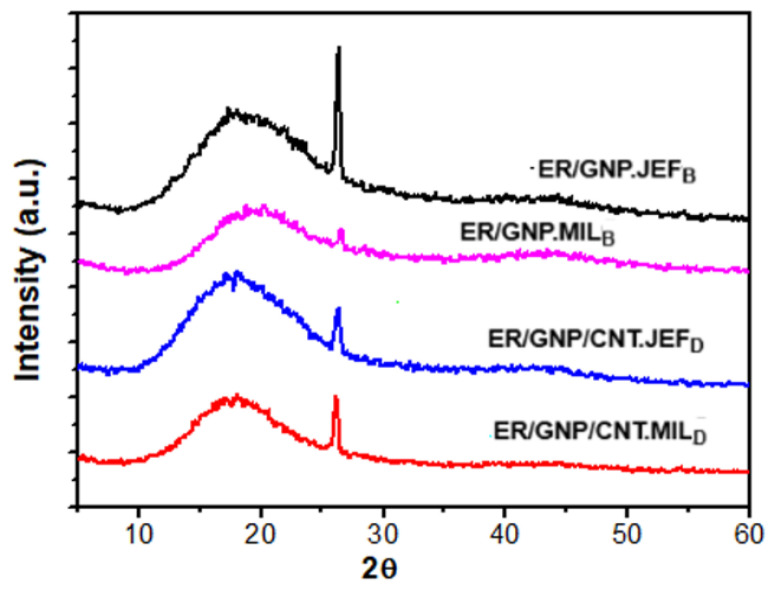
XRD profiles of the ER-based composites.

Figure 3 presents the XRD profiles of the composites cured with MIL and Jeffamine. The XRD diffractogram of neat GNP is shown in Figure S2. Neat GNP exhibits an intense diffraction peak at 2θ = 26.43° (d-spacing: 03.37 Å) and a small peak at 2θ = 44.6°, related to the (002) and (100) crystallographic planes of the graphitic structure, respectively. The nanocomposites present a wide diffraction peak from 10° to 28°, attributed to the scattering of the cured epoxy molecules, along with the sharp peak at 2θ = 26.43° related to the graphite planes. This second peak suggests that the GNP layers could not be completely exfoliated. Nevertheless, the intensity of the peak related to the graphite layers varied, indicating some degree of exfoliation. In this context, the ER/GNP.JEF_B_ displayed the more intense peak, indicating a lower degree of graphite exfoliation. On the other hand, the peak intensity corresponding to the GNP in the composite cured with MIL (ER/GNP.MIL_B_) significantly decreased, suggesting an increase in the exfoliation degree promoted by the presence of MIL as the curing agent. This result confirms the efficiency of the ionic liquid as a dispersing agent for the GNP filler. The presence of CNT in the composites cured with both Jeffamine and MIL decreased the peak intensity, suggesting an increase in the exfoliation due to the presence of CNT. This behavior confirms the role of MIL as dispersing agent for the carbonaceous filler, and as the curing agent for the epoxy matrix.

**Figure 4:**
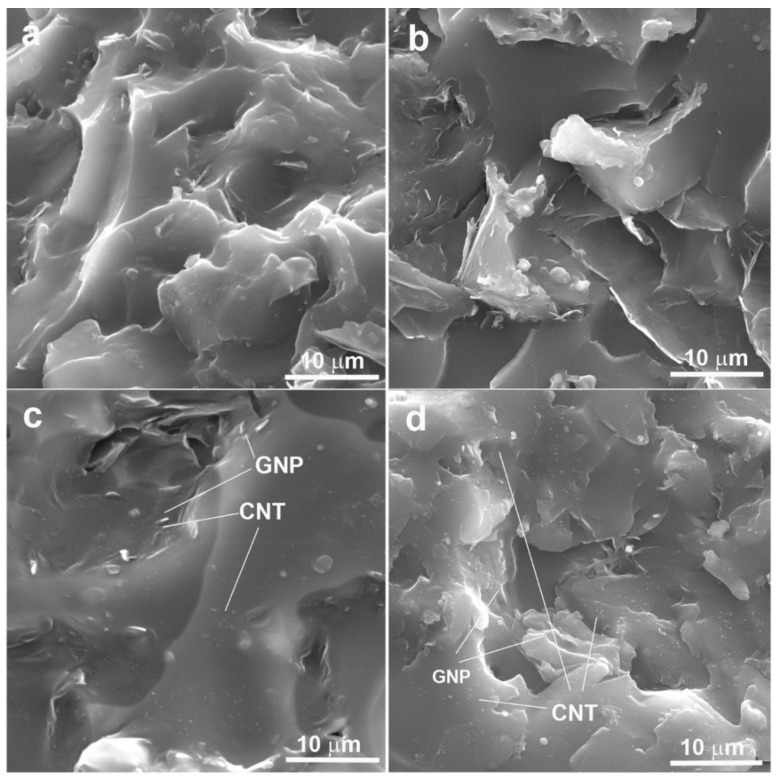
SEM images of (**a**) ER/GNP.MIL_B_; (**b**) ER/GNP.JEF_B_; (**c**) ER/GNP/CNT.MIL_D_, and (**d**) ER/GNP/CNT.JEF_D_.

The effect of MIL on the dispersion of the filler within the epoxy network was evaluated using SEM and TEM microscopy. Figure 4 compares SEM images for ER/GNP and ER/GNP/CNT nanocomposites cured with MIL and Jeffamine D230. The ER/GNP.MIL_B_ composite (2.2 wt% GNP) (Figure 4a) displayed more dispersed GNP platelets compared to similar system ER/GNP.JEF_B_ (Figure 4b). This result indicates that MIL not only serves as the curing agent but also acts as a dispersing agent for the GNP, as was also observed in the XRD analysis. The ER/GNP/CNT.MIL_D_ system, cured with MIL (Figure 4c), showed even better filler dispersion. In this image, some white spots, attributed to CNT, are observed between the GNP platelets, forming the so-called “bridge” that enhances the conducting pathway. The hybrid system cured with Jeffamine also exhibited some thin layers of GNP (Figure 4d). However, GNP aggregates were also present.

The EDS spectra and mapping of the cross-section of ER/GNP.MIL_B_ and ER/GNP/CNT.MIL_D_ composites are exhibited in Figure S3 of the Supplementary Materials. The presence of P, Cl, and Fe in the EDS profiles of both systems revealed the presence of the MIL. Moreover, the elemental mapping confirms the homogeneous distribution of the MIL throughout the sample.

**Figure 5:**
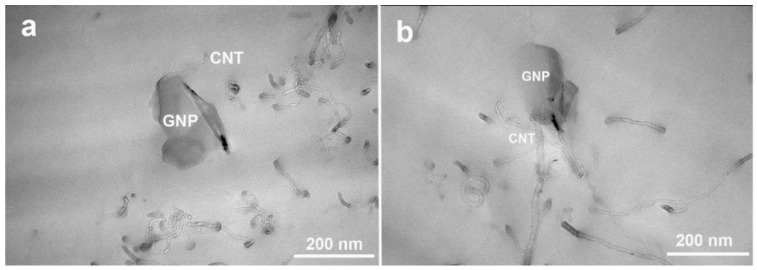
TEM images of (**a**) ER/GNP/CNT.JEF_D_ and (**b**) ER/GNP/CNT.JEF_D_ composites.

The TEM images of ER/GNP/CNT.MIL_D_ and ER/GNP/CNT.JEF_D_ are displayed in Figure 5. In both images, it is possible to observe the presence of CNT interacting with the GNP platelets, confirming that CNT acts as a bridge favoring the dispersion of GNP. Furthermore, the CNTs are well dispersed in these systems, which explain the superior conductivity of the hybrid composites.

### 2.4. Magnetic Properties of Composites Cured with MIL

**Figure 6:**
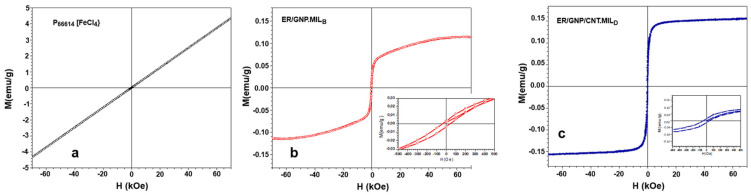
Hysteresis curves at 300 K for the ionic liquid P_66614_[FeCl_4_] (MIL) (**a**), and the composites ER/GNP.MIL_B_ (**b**) and ER/GNP/CNT.MIL_D_ (**c**), as a function of the applied magnetic field. The inset in (**b**) and (**c**) show the coercivity of about 40 Oe of theses composites.

The magnetic properties of neat MIL (P_66614_[FeCl_4_]) and the ER-based composites cured with MIL (ER/GNP.MILB and ER/GNP/CNT.MILD) are illustrated in Figure 6. The magnetization curve for the neat MIL (Figure 6a) presented a linear dependence of the magnetization with the applied magnetic field, indicating paramagnetic behavior [64]. The magnetic susceptibility of (P_66614_[FeCl_4_]) was determined from the slope of the fitted line as 6.16 × 10^−5^ emu/g. Thus, the Curie–Weiss law described in Equation (1) can be applied to determine the main magnetic parameters:

(1) $$\chi^{-1}=\frac{T}{C}-\frac{\theta_{P}}{C}$$

where *χ* is the magnetic susceptibility, *C* is the Curie constant, and *θ_P_* is the paramagnetic Weiss temperature that is proportional to the intensity of the magnetic moment interactions [65].

The thermal variation of the inverse magnetic susceptibility is presented in Figure S4A of the Supplementary Materials. From this Figure, the *C* and *θ_P_* values corresponding to 0.019 emu K/g OE and −8 K were obtained, which are in agreement with the literature [66]. The negative value of *θ_P_* indicates antiferromagnetic coupling between the magnetic moments. Figure S4B exhibits the thermal variation of magnetization for the neat MIL under a magnetic field of 30,000 Oe. The magnetization increased with the temperature up to 8 K, after which it decreased, following a paramagnetic thermal transition. The reduction in moment for temperatures below 8 K can be associated with the change in moment direction toward an antiparallel alignment. Since *θ_P_* is proportional to the intensity of magnetic interactions, the small magnitude of *θ_P_* (8 K) indicates that these interactions are very weak, as expected for diluted [FeCl_4_]^−^ magnetic anions in an ionic liquid matrix.

The hysteresis curves at 300 K for the ER composites are presented in Figure 6b,c, and exhibited the typical S-shape magnetic hysteresis. The magnetization profile of ER/GNP.MILB (Figure 6b) agrees with a mixture of ferromagnetic contribution (evidenced by the saturation) and a slight paramagnetic contribution (indicated by the small linear contribution before saturation). Regarding the composite loaded with the hybrid filler (ER/GNP/CNT.MILD), the curve indicates that the saturation was achieved at low fields, suggesting a “soft” ferromagnetic behavior. The magnetization saturation for the ER/GNP.MIL_B_ and ER/GNP/CNT.MIL_D_ corresponded to 0.113 emu/g and 0.155 emu/g, respectively. These values are significantly lower than those usually observed for other magnetic materials, due to the very low amount of FeCl_4_ in the materials. Liu et al. [67] also reported the magnetization hysteresis of epoxy containing the Fe_3_O_4_/GNP hybrid. The presence of 0.5% of filler displayed saturation magnetization of 0.63 emu/g. The coercitivity (Hc) of the ER/GNP.MIL_B_ and ER/GNP/CNT.MIL_D_ composites is illustrated in the insert of Figure 6b and Figure 6c, respectively. In both composites, a coercitivity of around 40 Oe was observed, which is similar to some other systems in the literature as epoxy nanocomposites loaded with ternary filler involving RGO/polyaniline/Fe_2_O_3_ [68].

### 2.5. Dynamic Mechanical Analysis and Thermal Stability

**Figure 7:**
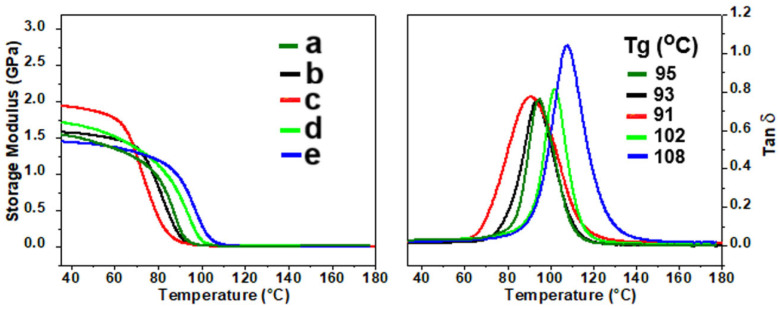
Storage modulus and tan delta against temperature for the ER composites containing GNP/CNT hybrid fillers: a—neat ER network; b—ER/GNP.JEF_B_; c—ER/GNP.MIL_B_; d—ER/GNP/CNT.JEF_D_ and e—ER/GNP/CNT.MIL_D_.

The effect of the curing agent on the main dynamic mechanical properties of ER composites is illustrated in Figure 7 in terms of storage modulus (E′) and tan delta (δ) against temperature. Regarding the ER/GNP systems, the one cured with MIL displayed higher E’ in the glassy region indicating better rigidity. However, the values of glass transition temperature (Tg), determined from the maximum of tan delta peak, for the ER/GNP systems cured with MIL and Jeffamine were slightly lower than the neat epoxy network, which can be attributed to the presence of free volume at the filler–matrix interface causing an increase in the epoxy chain mobility. Moreover, the E′ in the rubbery region was slightly lower than that of neat epoxy, indicating a decrease in the crosslinking density.

The hybrid composite cured with MIL displayed slightly lower E’ value, in both glassy and rubbery regions, than that observed for the system cured with Jeffamine, probably due to the plasticizing effect of MIL. However, the Tg of the system cured with MIL (ER/GNP/CNT.MIL_D_) displayed the highest Tg value, due to the better distribution of the filler within the epoxy matrix, thus decreasing the free volume at the interface.

**Figure 8:**
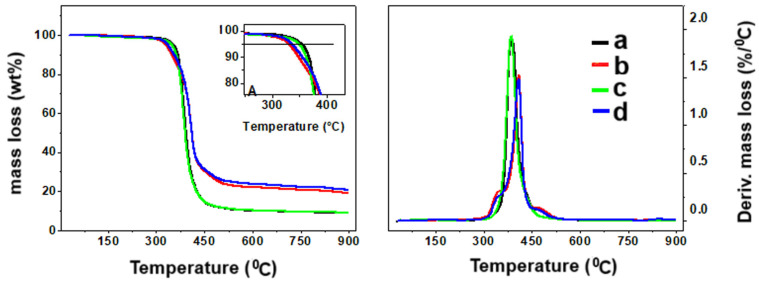
TGA and DTG thermograms of a—ER/GNP.JEF_B_; b—ER/GNP.MIL_B_; c—ER/GNP/CNT.JEF_D_ and d—ER/GNP/CNT.MIL_D_.

The thermo-gravimetric analysis of the ER composites with different curing systems is illustrated in Figure 8. The main decomposition parameters are also summarized in Table S1. All samples were thermally stable until 300 °C. At 5 wt% of mass loss, the composites cured with MIL presented a lower decomposition temperature due to the decomposition of the ionic liquid moiety. This feature is clearly observed in the DTG curves. However, considering the maximum decomposition temperature, the composites cured with MIL exhibited the shift of the temperature towards higher values, indicating that the systems cured with phosphonium based IL (MIL) presented better thermal stability. The thermal decomposition of the composites resulted in a great amount of residue due to the presence of carbonaceous fillers. In these cases, the amount of residue for the samples cured with MIL presented a greater amount of char due to the presence of iron in the MIL moiety.

### 2.6. Electromagnetic Interference Shielding Effectiveness and Absorbing Properties

**Figure 9:**
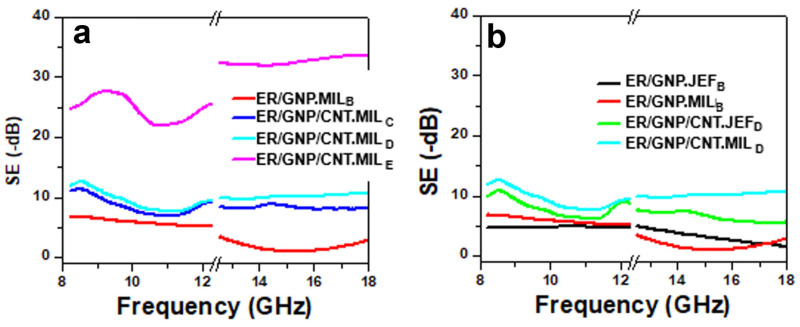
EMI SE in the X- and Ku-bands for ER composites. (**a**) ER/GNP and ER/GNP/CNT composites cured with MIL; (**b**) comparison between the composites cured with MIL and with Jeffamine D230.

The electromagnetic interference shielding effectiveness (EMI SE) and the calculated reflection loss (RL) were evaluated in the X-band (8–12 GHz) and Ku-band (12–18 GHz), due to the potential applications in these frequency ranges. The X-band (8–12 GHz) is used in high precision radars (military, earth exploration, and weather) and tactical communication, whereas the Ku-band (12–18 GHz) is useful for military satellite communication, radionavigation/radiolocation, broadcasting, and in-flight Wi-Fi [69]. The effect of the CNT content on the EMI SE of ER/GNP hybrid composites cured with MIL is illustrated in Figure 9a. The composite containing only GNP (2.2 wt%) (ER/GNP.MIL_B_) displayed relatively low EMI SE (6.8–5.5 dB) in the X-band frequency range due to the small amount of filler in this sample and low conductivity, resulting in little quantities of free charge carrier to interact with the electromagnetic (EM) radiation. Increasing the amount of CNT in the hybrid also increased the EMI SE in both X- and Ku-band, due to the rise in electrical conductivity and interfacial polarization properties [70]. The EMI SE values reached 27.64 dB at around 9 GHz (X-band) and 33.20 dB at 17 GHz (Ku-band) for the composite containing GNP/CNT = 2.2:2.2 wt% (ER/GNP/CNT.MIL_E_).

The effect of the curing system on this property was also compared in Figure 9b. The characteristics of the curing agent did not exert great influence on the system loaded with 2.2 wt% GNP due to the low conductivity of both materials. However, better EM wave attenuation was observed for the ER/GNP/CNT.MIL_D_ sample cured with MIL in both X- and Ku-frequency ranges. This behavior is attributed to the higher conductivity and to the presence of the magnetic ionic liquid that can also interact with EM radiation through the magnetic dipoles.

**Figure 10:**
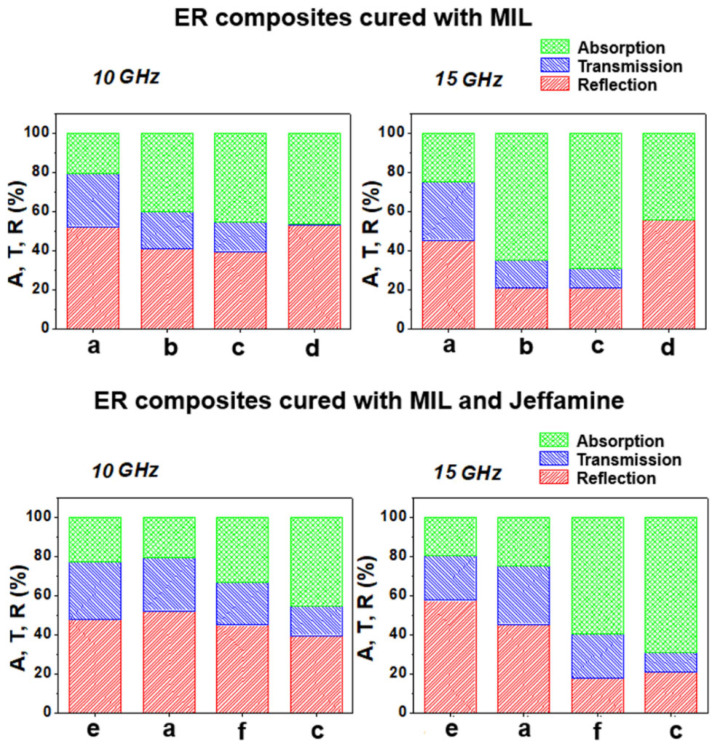
Percentage of absorption, transmission, and reflection of ER hybrid composites a—ER/GNP.MIL_B_; b—ER/GNP/CNT.MIL_C_; c—ER/GNP/CNT.MIL_D_; d—ER/GNP/CNT.MIL_E_; e—ER/GNP.JEF_B_; and f—ER/GNP/CNT.JEF_D_, at frequencies of 10 GHz (X-band) and 15 GHz (Ku-band).

The main mechanisms involved in the EM SE of a material are the reflection of the radiation at the surface of the material and the absorption of the radiation within the material [1,2,3,4]. Figure 10 shows the percentage of absorption (A), transmission (T), and reflection (R) for the different systems at frequencies of 10 GHz and 15 GHz, which correspond to the mid-range of the X- and Ku-bands, respectively. The data were determined from the scattering parameters, according to Equations (2)–(4):

(2) $$PR={\left(S_{11}\right)}^{2}$$

(3) $$PT={\left(S_{21}\right)}^{2}$$

(4) PA=1−Pr−PT

Table S2 of the Supplementary Materials also summarizes these results. The percentage of the EM radiation transmission remained low for most compositions, indicating good EMI SE of these materials. The percentage of transmitted EMW for the ER/GNP/CNT.MIL_E_ sample was negligible due to the high conductivity and greater amount of free charge carrier in this sample. This feature also caused an increase in the reflection contribution to the overall EMI SE. The contribution of the absorption to the total SE increased in the hybrids containing GNP/CNT = 2.2:0.2 and 2.2:0.4 wt% and this behavior was more pronounced in the Ku-band.

The EMI SE of materials is influenced by electrical conductivity, as well as dielectric and magnetic properties. The effect of the CNT content on the permittivity (ε) and permeability (µ) of ER/GNP/CNT composites cured with MIL are shown in Figure S5. The real (ε′) and imaginary (ε″) parts of the complex permittivity represent the dielectric properties, while the real (µ′) and imaginary (µ″) parts of the complex permeability represent the magnetic properties of the material. The complex permittivity (ε′ and ε″) values were significantly higher than the permeability (µ′ and µ″) values, indicating greater contribution of the dielectric properties to the EMI SE. The real and imaginary parts of permeability present some fluctuation along the frequency range studied due to variation in the interaction of the EMW with the material and the heterogeneous distribution of the magnetic entities. Although the ionic liquid has a magnetic character, the magnetization values of the composites are low, as discussed before, and explain the low values of magnetic permeability. The addition of a small amount of CNT in the system did not exert significant influence on the dielectric properties. However, for the ER/GNP/CNT.MIL_E_ composite, a great increase in both real and imaginary parts of complex permittivity was observed, due to the higher number of interfaces and space charge polarization [71]. Both real and imaginary parts of permeability were low in all frequency ranges studied. This behavior can be attributed to the low magnetization response of the composites, as observed by the magnetization measurements, due to the low amount of the magnetic ionic liquid. The slight fluctuation of the µ′ and µ″ values may be attributed to the magnetic nature of the IL.

An effective shielding material should present the absorption as the dominant mechanism, to minimize the EMW interference caused by the reflection on the neighboring electronic devices. The microwave-absorbing (MWA) performance of a material can be evaluated by a parameter known as reflection loss (*RL*) and the effective absorption bandwidth (EAB), that is, a bandwidth with *RL* < −10 dB, which corresponds to an EMW attenuation of 90%. A good absorbing material should present negative RL values as low as possible (higher attenuation) in a specific frequency and a broad EAB. According to the transmission line theory, *RL* can be calculated from the input impedance (*Z_in_*) of the material and the free space impedance (*Z*_0_ = 377 Ω), according to Equation (5), which in turn depends on the relative complex permeability (μ_r_), complex permittivity (ε_r_), the measured frequency (f), speed of light (c), and the sample thickness (d), according to Equation (6) [72,73]:

(5) $$RL\left(dB\right)=-20log\left|\frac{Z_{in}-Z_{0}}{Z_{in}+Z_{0}}\right|$$

(6) $$Z_{in}=\sqrt{\frac{\mu_{r}}{\varepsilon_{r}}}tanh\left(j\frac{2\pi fd}{c}\sqrt{\varepsilon_{r}\mu_{r}}\right)$$

Materials that are effective absorbers have impedance matching values close to one (*Z_in_*/*Z*_0_ ≈ 1) and minimal return loss (*RL*). Under these conditions, electromagnetic radiation can penetrate the material without reflection [74].

**Figure 11:**
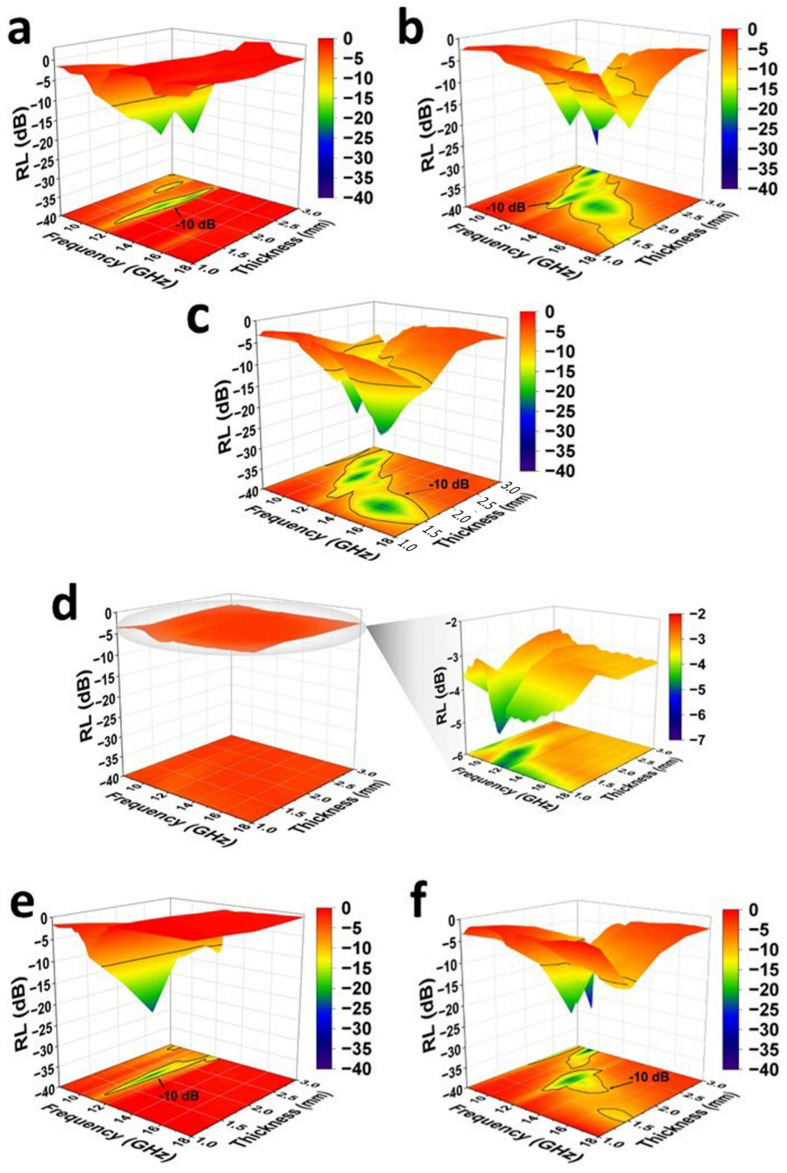
Calculated reflection loss (RL) as function of thickness and frequency for the composites (**a**) ER/GNP.MIL_B_; (**b**) ER/GNP/CNT.MIL_C_; (**c**) ER/GNP/CNT.MIL_D_; (**d**) ER/GNP/CNT.MIL_E_; (**e**) ER/GNP.JEF_B_; and (**f**) ER/GNP/CNT.JEF_D_.

Figure 11 shows the 3D plots of calculated reflection loss (*RL*) of ER/GNP/CNT composites, as a function of frequency and thickness. For better detail, the minimum *RL* values, the peak frequency, and the effective absorption bandwidth (EAB) are also summarized in Table 1. The matching thickness corresponds to the thickness that exhibits the highest EMW attenuation (minimum *RL*) and the broader EAB, which is the largest frequency range with *RL* < −10 dB (EMW attenuation higher than 90%). The composite loaded with 2.2 wt% of GNP presented the minimum *RL* of around −30 dB (99.9% attenuation) with 1.5 mm thickness, regardless of the curing system. The greatest EAB values were achieved with thickness corresponding to 2.5 (EAB = 1.69 GHz) and 3.0 mm (EAB = 1.81 GHz) for the systems cured with MIL and Jeffamine, respectively. Although the *RL* values in these systems correspond to a great EMW attenuation, the EAB values indicate a narrow frequency range for the attenuation.

The addition of 0.2 wt% of CNT in the system (ER/GNP/CNT.MIL_C_) led to an improvement of the EMW attenuation with 3 mm thickness. At this thickness, RL and EAB values corresponding to −33.07 dB and 2.14 GHz were achieved, respectively. The best compromise between RL and EAB values was observed for the ER/GNP/CNT.MIL_D_ composite with 1.5 mm thickness, where RL of −23.41 dB (higher than 99% attenuation) and an EAB of 5.18 GHz were observed. The similar composite cured with Jeffamine (ER/GNP/CNT.JEF_D_) presented the minimum RL (−28.59 dB) with 3 mm thick sample, but the larger EAB (3.65 GHz) was observed for the composite with 2.0 mm thickness. Thus, using hybrid filler and MIL as the curing agent was interesting to develop composites with outstanding EMW attenuation by absorption mechanism with relatively small thickness. This behavior is important for reducing density and cost and improving processability.

Table 2 compares the EMI SE and RL results of some epoxy nanocomposites reported in the literature with the data obtained in this work. The EMI SE value observed in the present work, for the epoxy nanocomposite containing GNP/CNT (2.2:2.2 wt%) of hybrid filler and cured with MIL, was superior to those found in most of the cited systems, with a lower amount of filler. Moreover, the RL and EAB values were quite favorable if one takes into consideration the simple, scalable, and solvent-free method used in this work.

## 3. Experimental

### 3.1. Materials

Epoxy resin (ER) based on bisphenol A diglycidyl ether (Araldite^®^ GY 260) (epoxide equivalent weight = 182–192 g/mol; viscosity (25 °C) = 12–16 Pa·s) was purchased from Huntsman Advanced Materials (São Paulo, Brazil). Trihexyltetradecylphosphonium chloride (P_66614_.Cl; ≥95%) (Cyphos^®^ IL101) and poly(propylene glycol) bis(2-aminopropyl ether) (Jeffamine^®^ D-230) were purchased from Sigma-Aldrich (São Paulo, Brazil). A multi-walled carbon nanotube NC7000 (CNT) (average diameter = 9.5 nm; average length = 1.5 μm and carbon purity = 90%) was purchased from Nanocyl S.A (Sambreville, Belgium). Graphene nanoplatelets Grade M (surface area = 150 m^2^/g; particle size < 5 μm) (xGnP^®^) were purchased from Nano Xplore, Inc. (Montreal, QC, Canada). Anhydrous ferric chloride (FeCl_3_) was purchased from Sigma-Aldrich (São Paulo, Brazil).

### 3.2. Synthesis of Magnetic Ionic Liquid (MIL)

**Figure 12:**
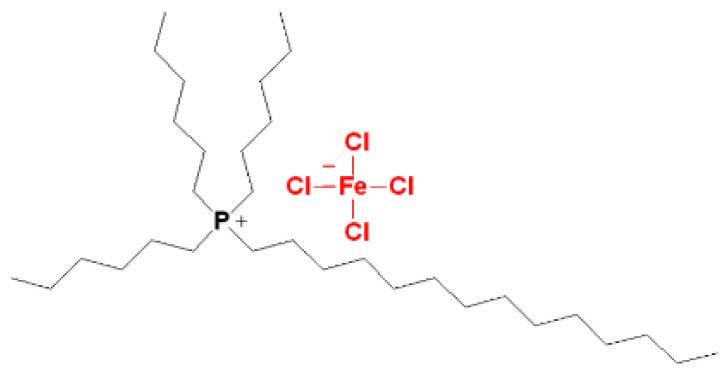
Structure of trihexyl(tetradecyl)-phosphonium tetrachloroferrate (P_66614_[FeCl_4_]).

The synthesis of the magnetic ionic liquid (MIL), trihexyl(tetradecyl)phosphonium tetrachloroferrate (P_66614_[FeCl_4_]), was performed according to previous works [53,78]. For the synthesis, P_66614_.Cl and anhydrous FeCl_3_ were mixed in equimolar proportions of 0.02 mol and stirred for 24 h at 60 °C under a nitrogen atmosphere. Then, MIL was obtained as a brown viscous oil (yield = 95%; melting point = −6.5 °C). Figure 12 illustrates the structure of the phosphonium ionic liquid.

### 3.3. Preparation of Epoxy Resin (ER) with Hybrid Fillers

For the preparation of ER composites cured with MIL, GNP and CNT fillers were first mixed with MIL and ER in a Cryomill ball mill from Retsch, using the following parameters: 4 cycles of 15 min each at a frequency of 30 Hz, with 3 solid steel balls of 1.0 mm of diameter. After mixing, the system was transferred to a speed mixer operating at 3500 rpm for 5 min to remove possible bubbles and poured into silicone molds, with appropriate dimensions for each characterization. The curing parameters followed protocols described in the literature: 1 h at 130 °C; 3 h at 200 °C; 2 h at 220 °C [53]. The amount of MIL was fixed as 10 phr (part per hundred part of resin), which was enough to achieve good curing level and glass transition temperature, as well as a reasonable response in terms of microwave-absorbing properties, according to previous work [53].

The ER composites cured with Jeffamine were prepared by mixing GNP and CNT with the ER in the Cryomill ball mill, using the same mixing protocol described before. Then, Jeffamine D230 (32 phr, according to the manufacturer) was added and the system was transferred to the speed mixer as previously described. The compositions are summarized in Table 1. Although the amounts of hardener and additives in epoxy formulations are commonly expressed in phr, Table 3 displays the composition of each composite in phr and percentage, to unify the units of measurements with those normally used for conductive polymeric composites. The amount of GNP was fixed as 2.5 phr to emphasize the influence of the presence of low amount of CNT on the electrical properties. However, the amount of filler presents some variation when using percentage as the unit.

### 3.4. Characterization

The rheological characterization of the ER/hybrid systems was carried out in a Discovery DHR 1 rheometer (TA Instruments Inc.—New Castle, DE, USA) at 25 °C, using parallel plate geometry (25 mm) with a gap of 1 mm. The viscoelastic properties were determined through a frequency sweep of 0.1 to 100 Hz with 0.1% of deformation.

Dynamic mechanical analysis (DMA) was performed in a Q800 equipment (TA Instruments Inc.—New Castle, DE, USA), equipped with a single cantilever clamp using sample dimensions of 35.0 D7 12.5 × 3.0 mm. The conditions of the analysis were as follows: frequency = 1 Hz; heating from 25 °C to 180 °C; heating rate = 3 °C/min; deformation = 0.1%.

The electrical conductivity was measured in an Autolab PGSTAT204 impedance analyzer from Metrohm (Herisau, Switzerland) in alternating current (AC) mode. The measurements were conducted at room temperature with discs of 25 mm of diameter and 1 mm thickness, in a frequency range from 10^−1^ Hz to 10^6^ Hz, with a voltage of 0.1 V. The samples were coated with a thin layer of gold to improve electrical contact.

The electromagnetic absorbing properties of the materials were measured in terms of shielding effectiveness (SE) and reflection loss (RL) in the microwave X-band (8.2–12 GHz) and Ku-band (12–18 GHz) ranges, using a VNA Vector Network Analyzer, model E5080B from Keysight Technologies (Chicago, IL, USA) with a rectangular waveguide. Samples with 3.0 mm thickness were used.

Scanning electron microscopy (SEM) was performed on a TESCAN model VEGA II microscope (Brno—Czechia) operating at 20 kV. The fractured surfaces of the epoxy composites were coated with a thin layer of gold before analyzing, using a secondary electron detector and energy dispersive X-ray (EDX) detector from Bruker Nano GmbH (Berlin, Germany), model XFlash 630 M.

The X-ray diffraction (XRD) patterns were obtained on a Rigaku Ultima IV diffractometer (Tokyo, Japan), using a continuous scanning mode in the range of 2θ from 5° to 60°, with a step size of 0.05° s^−1^, using the Cu-kα radiation at 40 kV and 40 mA.

The magnetic measurements were performed using a Quantum Design MPMS3 magnetometer (San Diego, CA, USA) (applying magnetic fields up to 7 Tesla and temperature ranging from 2 K to 400 K). The available sample holders are designed for solid samples. For the liquid MIL, it was introduced into a quartz tube with a diameter smaller than that of the standard plastic sample holder (~6 mm diameter). To prevent the vacuum in the measurement chamber from aspirating the liquid, the quartz tube was sealed using a flame generated from an acetylene–air mixture.

Transmission electron microscopy images were obtained from a Hitachi model HT 7800 (Tokyo, Japan) operating at 100 kV. The samples were ultramicrotomed in a Leica equipment with a thickness of around 60 nm.

## 4. Conclusions

Epoxy nanocomposites containing GNP/CNT hybrid filler were prepared in the presence of a magnetic ionic liquid, which served a dual role as both a curing agent for the epoxy matrix and a dispersion agent for the fillers. A significant increase in electrical conductivity was observed when combining 2.2 wt% of GNP with 0.2 wt% of CNT, compared to the ER/GNP binary composites containing 2.2 and 8.3 wt% of GNP. Furthermore, the use of MIL as the curing agent resulted in epoxy nanocomposites (ENCs) with higher conductivity and superior EMI SE compared to conventional aliphatic amine. This indicates that the ionic liquid not only dispersed the fillers efficiently but also facilitated the formation of a conductive pathway.

Additionally, the systems cured with MIL demonstrated greater contribution of the absorption mechanism in their EMI SE measurements, likely due to the presence of magnetic moiety in the ionic liquid. The ENC containing 4.4 wt% of the hybrid filler (GNP/CNT = 2.2:2.2 wt%) exhibited an EMI SE of 33 dB, which surpasses several other epoxy-based systems reported in the literature. Moreover, a significant EMW attenuation response was observed, with a minimum RL of −23.4 dB and a large EAB of 5.18 GHz, for the 1.5 mm thick ENC containing 2.6 wt% of hybrid filler (GNP/CNT = 2.2:0.4 wt%). In summary, this study presents a cost-effective and scalable approach for developing materials with absorption-dominated EMI shielding properties, since commercial materials such as GNP and CNT were employed in low amounts to decrease cost and maintain a good processability. Furthermore, the ball-milling technique used for mixing the ingredients does not require solvent, making large-scale production easier. Finally, the high microwave-absorbing properties in a large frequency range make this material suitable for civilian and military applications.

## Figures and Tables

**Table 1:** Calculated RL and effective absorption bandwidth (EAB) of the composites.

Composites	Thickness (mm)	RL (dB)	Frequency (GHz)	EAB (GHz)
Effect of the CNT content (samples cured with MIL)				
ER/GNP.MIL_B_	1.0	−8.93	12.97	-
	1.5	−30.41	12.94	1.24
	2.0	−22.71	12.61	1.46
	2.5	−22.26	11.99	1.69
	3.0	−10.68	8.27	0.32
ER/GNP/CNT.MIL_C_	1.0	−3.58	11.88	-
	1.5	−15.52	18.00	1.96
	2.0	−21.56	11.92	1.16
	2.5	−18.35	10.97	2.33
	3.0	−33.07	9.99	2.14
ER/GNP/CNT.MIL_D_	1.0	−5.86	18.00	-
	1.5	−23.41	15.03	5.18
	2.0	−23.69	11.10	2.31
	2.5	−20.55	10.01	2.10
	3.0	−10.80	9.21	0.48
ER/GNP/CNT.MIL_E_	1.0	−5.03	11.75	-
	1.5	−4.91	10.83	-
	2.0	−3.76	10.62	-
	2.5	−3.39	16.99	-
	3.0	−3.35	16.99	-
Effect of the CNT content (samples cured with Jeffamine)				
ER/GNP.JEF_B_	1.0	−11.49	13.24	0.17
	1.5	−30.74	13.19	1.36
	2.0	−24.17	11.04	1.63
	2.5	−12.09	14.77	1.40
	3.0	−13.51	14.23	1.81
ER/GNP/CNT.JEF_D_	1.0	−4.06	11.81	-
	1.5	−11.64	17.10	2.07
	2.0	−23.28	11.94	3.65
	2.5	−15.75	8.20	1.64
	3.0	−28.59	9.40	1.53

**Table 2:** Comparison data of EMI SE and RL of some epoxy nanocomposites reported in the literature.

Filler	Amount	Thickness (mm)	EMI SE (dB)/ Freq (GHz)	RL (dB)/ Freq (GHz)	EAB	Method	Ref.
GNP	30 (wt%)	2.5	20 (11)	−3.5 (10.5)	-	Mechanical mixing	[16]
nanographite	15 (wt%)	7.5	12 (11.6)	−10.5 (12)	1.3	Mechanical mixing	[16]
GNP	17 (wt%)	2	8 (8.4)	-	-	Sonication without solvent	[17]
GNP	30 (wt%)	1	23 (8.2)	-	-	high-shear speed mixer	[18]
RGO	15 (wt%)	3	21 (8–12)			Sonication with solvent	[19]
RGO	5 (wt%)	6	25 (12)	-	-	Mini-mechanical vortex mixer	[20]
GNP	15 (wt%)	3	-	−14 (18.9)	2.0	Sonication with solvent	[22]
GNP	15 (wt%)	2.5	5.85 (2)	-	-	Sonication without solvent	[24]
RGO/CNT	15 (wt%)	1.2	70 (12)	-	-	Chemical grafting of CNT onto GO/reduction	[37]
RGTO	1 (wt%)	1.9	-	−61.8 (13.5)	5.38	Expanded graphite/CNT oxidized together—sonication with solvent	[41]
TRGO/CNT (2.5:2.5)	5 (wt%)	2	12 (10)	-	-	Sonication with solvent	[43]
RGO-imid	1 (wt%)	3	-	−47.2 (12)	6.04	Sonication with solvent—modif. of RGO with imidazole	[75]
MFRGO	40 (wt%)	4	-	−25 (8.6)	1.2	Covalent functionalization of GO by imidazolium IL/reduction	[76]
Exfoliated graphite	44.7 (vol%)	4		−23.8 (11)	5.6	chemical treatment with maceration and microwave-assisted thermal exfoliation	[77]
GNP/CNT	2.6 (wt%)	3	10 (15–18)	-	-	Mixing in a ball-milling and cured with MIL	This work
GNP/CNT	2.6 (wt%)	1.5		−23.4	5.18	Mixing in a ball-milling and cured with MIL	This work
GNP/CNT	4.4 (wt%)	3	33.2 (17)			Mixing in a ball-milling and cured with MIL	This work

**Table 3:** Compositions in the hybrid composite matrix.

Sample Code	ER		MIL		GNP		CNT		Jef	
	(phr)	(wt%)	(phr)	(wt%)	(phr)	(wt%)	(phr)	(wt%)	(phr)	(wt%)
ER/GNP.MIL_A_	100	83.4	10.0	8.3	10.0	8.3				
ER/GNP.MIL_B_	100	88.9	10.0	8.9	2.5	2.2	-			
ER/GNP/CNT.MIL_C_	100	88.7	10.0	8.9	2.5	2.2	0.25	0.2		
ER/GNP/CNT.MIL_D_	100	88.5	10.0	8.9	2.5	2.2	0.5	0.4		
ER/GNP/CNT.MIL_E_	100	87.0	10.0	8.6	2.5	2.2	2.5	2.2		
ER/GNP.JEF_B_	100	74.3	-		2.5	1.9	-		32	23.8
ER/GNP/CNT.JEF_D_	100	74.1	-		2.5	1.8	0.5	0.4	32	23.7

ER/GNP.JEF_B_ Jef = Jeffamine D230.

## Data Availability

The datasets generated during and/or analyzed during the current study are available from the corresponding author on reasonable request.

## Supplementary Materials

The following supporting information can be downloaded at: https://www.mdpi.com/article/10.3390/molecules30050985/s1, Figure S1: Han plots of ER composites. (a) ER/GNP and ER/GNP/CNT cured with MIL (10 phr); (b) comparison between the composites cured with MIL(10 phr) and with Jeffamine D230 (32 phr); Figure S2: XRD profile of neat GNP; Figure S3: EDS data of the composites cured with MIL; Figure S4: Dependence of the (a) inverse magnetic susceptibility and (b) magnetization at 30,000 Oe with temperature of neat ionic liquid P_66614_[FeCl_4_]. Figure S5: Permittivity and permeability of ER composites; Table S1: Main thermal decomposition parameters observed from the TGA analysis; Table S2: Percentage of absorption, transmission and reflection related to the EMI SE measurements for ER composites loaded with GNP/CNT hybrid filler;.
