# Supplementary material for: Magnetic Ionic Liquid: A Multifunctional Platform for the Design of Hybrid Graphene/Carbon Nanotube Networks as Electromagnetic Wave-Absorbing Materials
Source: Molecules. 2025 Feb 20;30(5):985. doi: 10.3390/molecules30050985 (PMC11901776; doi:10.3390/molecules30050985)
Supplement: Supplementary file 1 [file molecules-30-00985-s001.zip › molecules-3427883-supplementary.pdf]

Magnetic Ionic Liquid: A multifunctional platform for the design of hybrid graphene/carbon nanotube networks as electromagnetic wave absorbing materials

Jean C. Carelo[1], Bluma G. Soares[1,2], Debora P. Schmitz[2], Ruan R. Henriques[2], Adriana A. Silva[3], Guilherme M. O. Barra[4], Vitoria M. T. S. Barthem[5], Sebastien Livi[6,*]*

[1] Universidade Federal do Rio de Janeiro, COPPE-PEMM, Centro de Tecnologia, Bl. F, 21941-598, Rio de Janeiro, RJ, Brazil

[2] Universidade Federal do Rio de Janeiro, Instituto de Macromoléculas, Centro de Tecnologia, Bl. J, 21941-598, Rio de Janeiro, RJ, Brazil

[3] Universidade Federal do Rio de Janeiro, Escola de Química, Centro de Tecnologia, Bl. E, 21941-909, Rio de Janeiro, RJ, Brazil

[4] Universidade Federal de Santa Catarina, Departamento de Engenharia Mecânica, Florianópolis, SC. Brazil.

[5] Universidade Federal do Rio de Janeiro, Instituto de Física, Centro de Tecnologia, Bl. A, 21941-909, Rio de Janeiro, RJ, Brazil

[6] CNRS, UMR 5223, Ingénierie des Materiaux Polymères, INSA-Lyon, F-69621, Villleurbanne, France

Corresponding authors: Bluma G. Soares – e-mail: bluma@metalmat.ufrj.br; Sebastien Livi – e-mail: sebastien.livi@insa-lyon.fr

Supporting Information

Figure S1. Han plots of ER composites. (A) ER/GNP and ER/GNP/CNT cured with MIL (10 phr); (B) comparison between the composites cured with MIL(10 phr) and with Jeffamine D230 (32 phr).

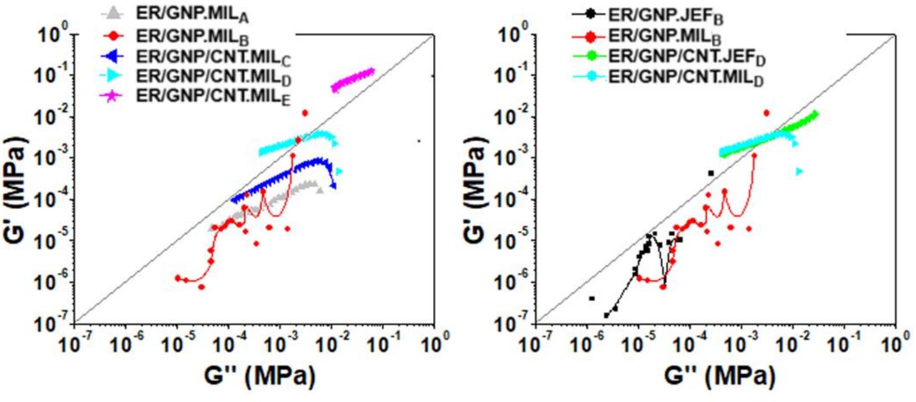

Figure S2. XRD profile of neat GNP

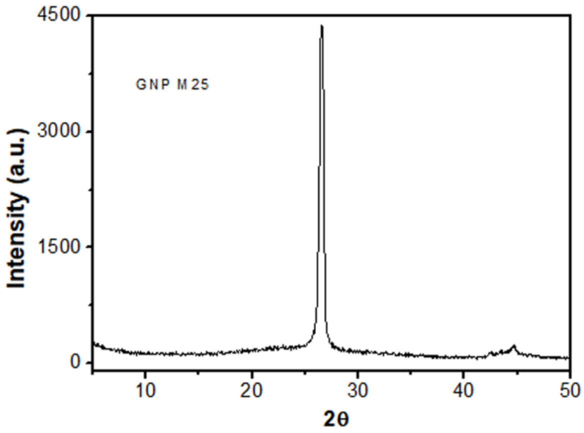

Figure S3. EDS data of the composites cured with MI

Analysis of ER/GNP.MIL$_B$

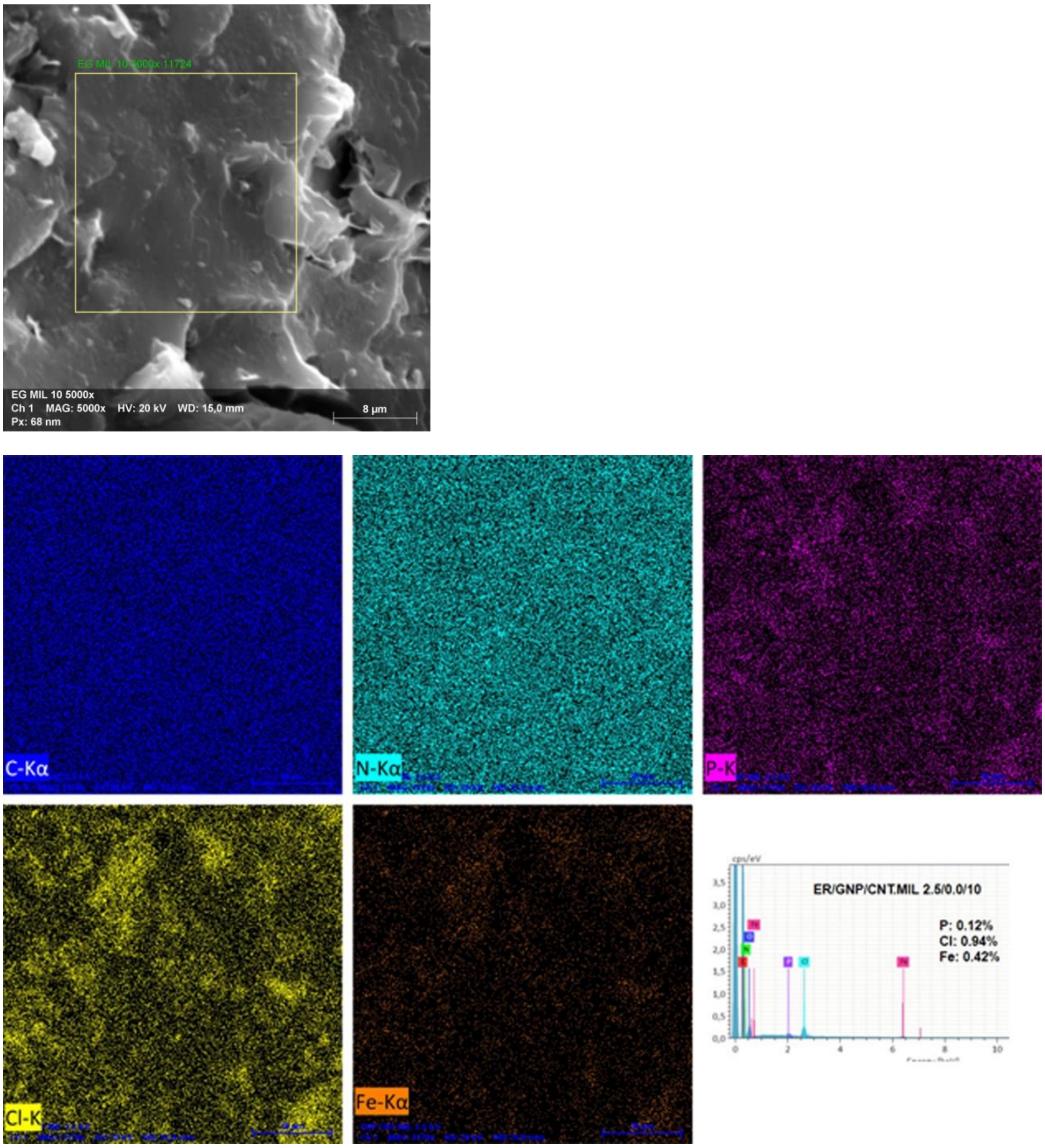

Analysis of ER/GNP/CNT.MIL$_D$

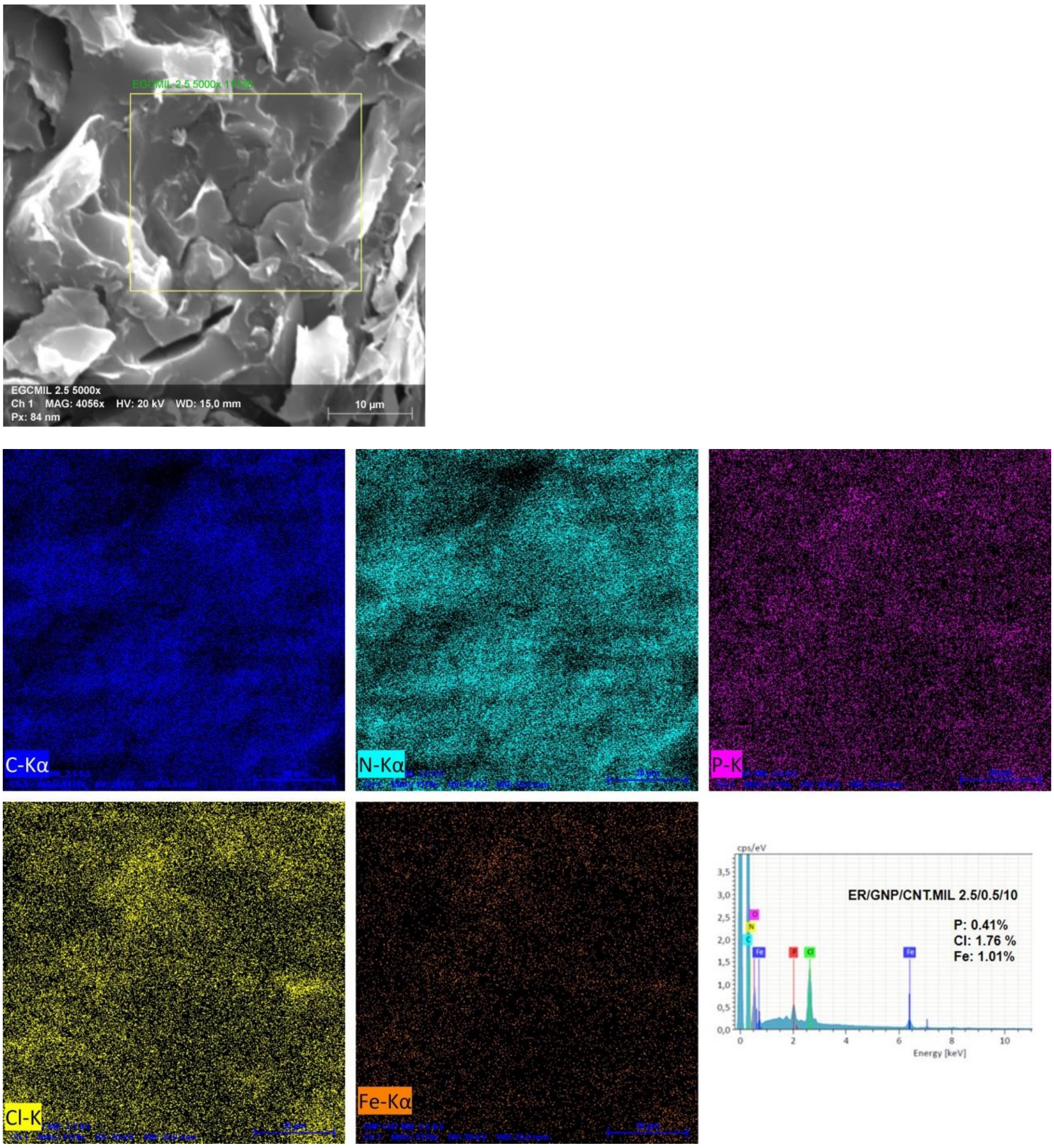

Figure S4. Dependence of the (A) inverse magnetic susceptibility and (B) magnetization at 30,000 Oe with temperature of neat ionic liquid $P_{66614}[FeCl_4]$.

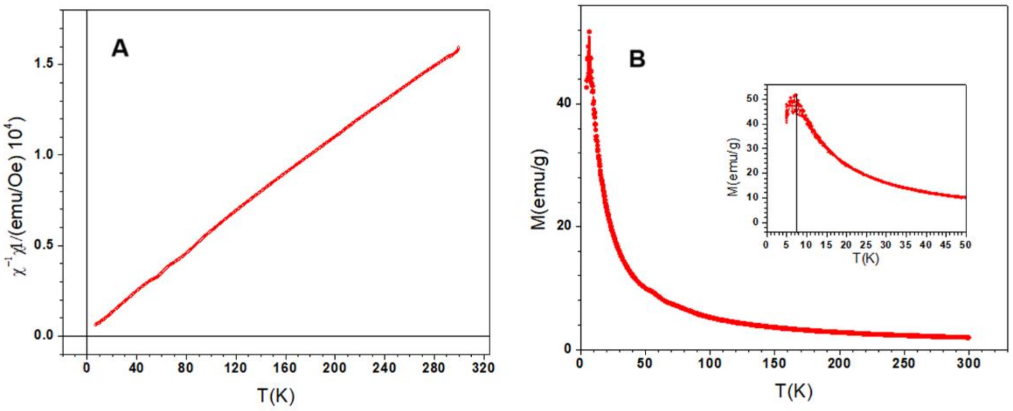

Figure S5. Permittivity and permeability of ER composites

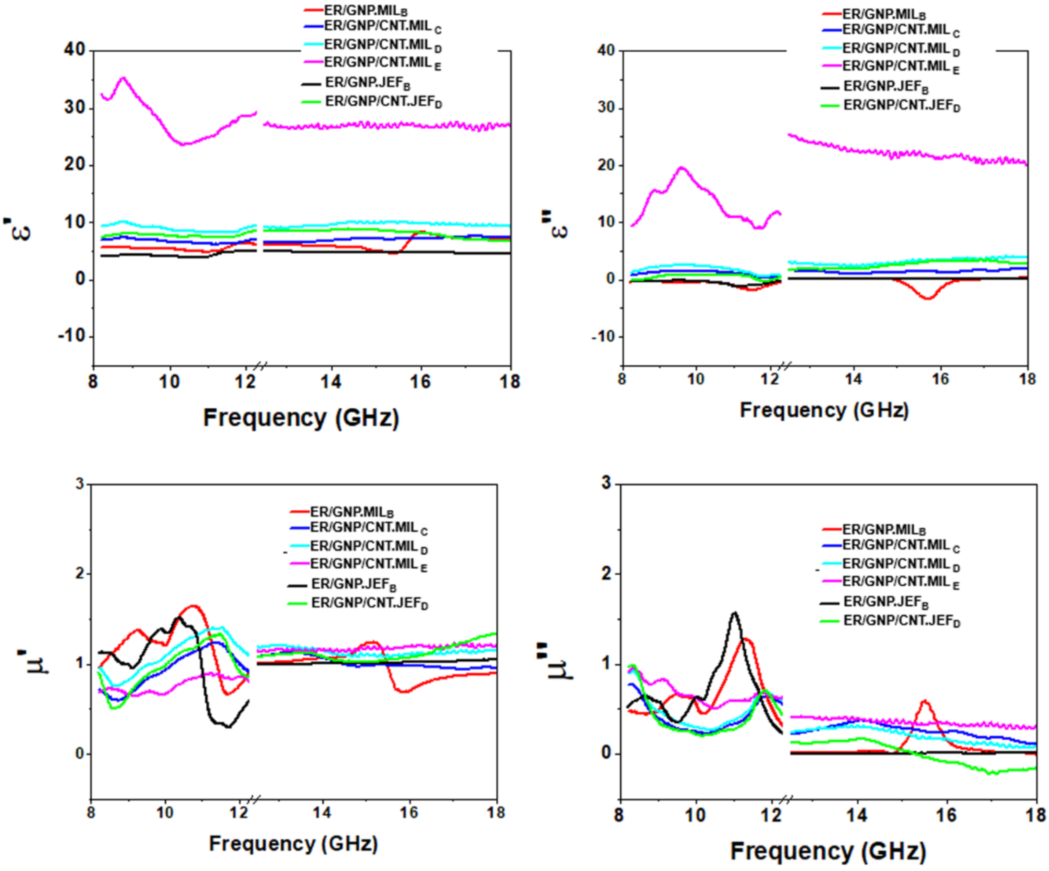

Table S1. Main thermal decomposition parameters observed from the TGA analysis

composite	T_{decomp} at 5 wt% mass loss (°C)	Maximum T_{decomp} (°C)	Residue at 800 °C (wt%)
ER/GNP.JEF$_B$	353	388	19
ER/GNP.MIL$_B$	329	408	22
ER/GNP/CNT.JEF$_D$	348	386	9
ER/GNP/CNT.MIL$_D$	335	408	9

Table S2. Percentage of absorption, transmission and reflection related to the EMI SE measurements for ER composites loaded with GNP/CNT hybrid filler

composites	10 GHz			15 GHz		
	R (%)	A (%)	T (%)	R (%)	A (%)	T (%)
ER/GNP.MIL$_B$	51	21	28	45	25	30
ER/GNP/CNT.MIL$_C$	40	40	20	21	65	14
ER/GNP/CNT.MIL$_D$	39	45	16	20	70	10
ER/GNP/CNT.MIL$_E$	49	50	1	54	44	2
ER/GNP.JEF$_B$	47	23	30	58	20	22
ER/GNP/CNT.JEF$_D$	44	33	23	18	60	22
